# Oral Health-Related Quality of Life in Patients after Stroke—A Systematic Review

**DOI:** 10.3390/jcm11051415

**Published:** 2022-03-04

**Authors:** Gerhard Schmalz, Simin Li, Dirk Ziebolz

**Affiliations:** 1 Department of Cariology, Endodontology and Periodontology, University Leipzig, Liebigstr. 12, 04103 Leipzig, Germany; dirk.ziebolz@medizin.uni-leipzig.de; 2 Stomatological Hospital, Southern Medical University, Guangzhou 510280, China; siminli1988@smu.edu.cn

**Keywords:** oral health, oral health-related quality of life, stroke, oral hygiene, quality of life

## Abstract

Objectives: Aim of this systematic review was to assess oral health-related quality of life (OHRQoL) of patients after stroke. Methods: The systematic literature search was performed on December 2021 based on PubMed, Medline, Web of Science, and Scopus, with the search terms: “oral health-related quality of life” AND stroke OR apoplexy OR ischemic stroke OR apoplectic insult. Articles exclusively examining patients after stroke and reporting a well-documented and valid OHRQoL measurement were included. Results: Out of 68 findings, 8 studies were included. The number of patients ranged between 31 and 549 individuals, mean age between 55.7 and 73.9 years, and 49–72% of individuals were male. Two studies included a healthy control group. Oral health parameters were rarely reported across studies. Five studies reported on the Oral Health Impact Profile (OHIP) 14 for OHRQoL, showing means between 2.87 and 33.0 in sum score. Three studies applied Geriatric Oral Assessment Index (GOHAI), with sum scores between 45.6 and 55.0. Only one study found worse OHRQoL in stroke patients compared to healthy controls. Two studies reported on an association between OHRQoL and general quality of life. Three studies found OHRQoL to be associated with different oral health parameters. Only one study found OHRQoL to be associated with stroke-related parameters. Conclusions: Patients after stroke show a reduced OHRQoL. Medical staff and caregivers should support oral hygiene and dental visits, to foster patients’ oral health and OHRQoL.

## 1. Introduction

Worldwide, stroke is a highly prevalent reason for long-term disability, morbidity, and death [1]. During the past years, many advances in preventive and therapeutic strategies for stroke have been made, which led to declining mortality rates; however, the overall global burden of stroke appears to increase continuously [2]. In this respect, the relevance of care for these individuals, especially during rehabilitative measures, is high and a field of increasing scientific interest [3]. Thereby, the health-related quality of life is an important aim and outcome of care for individuals after stroke [4]. It is known that quality of life is worse after stroke and positive or negative development is closely related to coping strategies, different personal and environmental factors, and psychological stressors like depression, anxiety, and posttraumatic stress disorder [4,5,6].

As one sub-aspect of the overall health-related quality of life, the construct of oral health-related quality of life (OHRQoL) has been established and widely recognized in the past decades [7,8,9]. Thereby, the OHRQoL reflects the potential influence of oral conditions, including dental, periodontal, and functional diseases, tooth loss, and various other pathologies on quality of life [8,10]. The OHRQoL includes different dimensions, whereby both functional and psychosocial sub-scales are available, which are potentially affected by different oral diseases [11,12]. Oral diseases are, in turn, common in stroke survivors; this is particularly related to physical, sensory, and cognitive complaints related to stroke, making oral health care challenging for those individuals [13]. Therefore, stroke survivors often have a higher burden of dental caries, periodontitis, and tooth loss, combined with a lower frequency of dental attendance [14]. Moreover, periodontitis has been reported to be a potential risk factor for stroke, whereby a risk ratio of 1.88 and 2.27 was found, depending on study design [15]. Those issues make the oral situation of patients after stroke a field of high scientific and clinical interest [14].

Taken together, the high morbidity and quality of life issues on the one hand, and the high oral disease burden of stroke survivors on the other hand, support the potential relevance of OHRQoL in these individuals. Previously, the OHRQoL of patients suffering from several general diseases was studied, including rheumatic diseases, renal insufficiency, organ transplantation, and Alzheimer disease [16,17,18,19]. Thereby, OHRQoL was repeatedly reported to not reflect physical oral health, but to be related to other (disease-related) issues in systemically diseased individuals [16,17,18,19]. For those reasons, the OHRQoL of patients after stroke appears an interesting and clinically relevant issue.

Accordingly, the aim of this systematic review was to investigate the OHRQoL of patients after stroke. Thereby, the potential associations of OHRQoL with oral health and general-disease related parameters were evaluated to reveal the most important influential factors on OHRQoL of those individuals. Based on the knowledge about patients with other systemic diseases, it was hypothesized that patients after stroke would show a slightly or moderately reduced OHRQoL, which is not primarily associated with their oral status.

## 2. Methods

The Preferred Reporting Items for Systematic Reviews and Meta-Analyses (PRISMA) formed the basis for this systematic review; thus, the whole process followed those guidelines [20].

### 2.1. PICO Question

Prior to systematic search, the following PICO (patients, intervention, comparison, outcome) question was formulated: “Is the OHRQoL of patients after stroke reduced compared to healthy individuals or available reference values?”. Accordingly, “patients” were individuals after stroke (irrespective of time since stroke). No “intervention” was defined, as the question would be answered by cross-sectional or case-control studies, respectively. “Comparison” was a healthy control group if applicable, otherwise reference values or cohorts with other systemic diseases. It was thereby decided to include studies without a healthy control as well, to allow inclusion of all studies reporting OHRQoL in these individuals. The “outcome” according to PICO was an OHRQoL measurement, whereby only well-documented and valid questionnaire-based assessments were considered. It was hypothesized that the OHRQoL of patients after stroke would be reduced with regard to healthy control individuals or reference values/available literature.

### 2.2. Eligibility Criteria

Several inclusion criteria were chosen for this systematic review:

- published before 30 November 2021;
- inclusion of patients after stroke, irrespective of their time since stroke and form of therapy or rehabilitation;
- reporting of a well-documented and valid OHRQoL measurement (especially questionnaire-based assessment);
- full-text in English language.

Articles which did not fulfil all of those criteria were excluded during the search and data extraction process.

### 2.3. Search Strategy

In December 2021 (date of literature search: 8 December 2021), two different and independent reviewers performed systematic literature search based on PubMed, Medline, Web of Science, and Scopus. The following search terms were applied: “oral health-related quality of life” AND “stroke” OR “apoplexy” AND “oral health-related quality of life” OR “ischemic stroke” AND “oral health-related quality of life” OR “apoplectic insult” AND “oral health-related quality of life”. Manual screening of the references of respective findings complemented the systematic search.

### 2.4. Data Extraction

Subsequently, following checking of the identified articles for their eligibility, qualitative data extraction was performed. The following information were assessed from the included publications:

- year of publication, number of participants, study type, age, gender, status, and time after stroke (time since stroke onset, form of current therapy, if applicable);
- inclusion of a healthy control group;
- information on oral health status, including tooth loss and dentures, dental as well as periodontal diseases, oral hygiene, and any further oral health parameters, if applicable;
- OHRQoL measurement and findings;
- associations between OHRQoL and quality of life, disease-related parameters, or oral health findings, if applicable;
- subscales of the OHRQoL, if applicable;

The information was double-checked by two independent reviewers. Only studies which exclusively reported on the OHRQoL of patients with stroke were considered within this review.

### 2.5. Quality Assessment

Based on the available 11-item checklist from the Agency for Healthcare Research and Quality (AHRQ) for cross-sectional studies, a quality appraisal of included investigations was performed [21]. Thereby, if answers were “no” or “unclear”, a score of 0 was added, while the answer “yes” was rated as 1 point for each question. Summarizing all 11 questions within one score was performed to estimate the quality of the studies. Based on this appraisal, scores of 0–3 indicated low, 4–7 indicated moderate, and 8–11 indicated high quality. As the whole systematic review process, the quality assessment was also performed by two independent examiners. Potential disagreements were discussed and resolved in the whole author group.

## 3. Results

### 3.1. Search Findings

**Figure 1:**
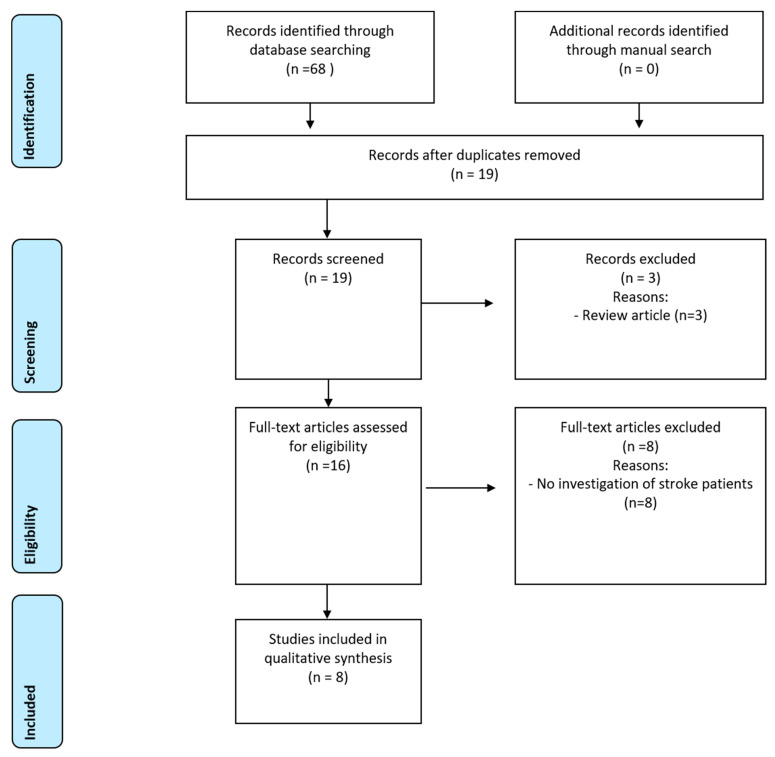
PRISMA diagram for systematic review process.

The systematic search revealed 68 results, from which 19 records were screened for eligibility. After exclusion of three review articles, 16 full texts were assessed. Half of those findings did not exclusively report on patients after stroke and were excluded, accordingly (Supplementary Materials Table S1). Finally, eight studies were included in qualitative analysis (Figure 1).

### 3.2. Characteristics of Included Studies

Five studies were monocentric cross-sectional studies [22,23,24,25,26], one had an observational design with 6 months follow-up [27], and two studies were randomized clinical trials [28,29]. The time since stroke varied between immediately after stroke and more than 10 years after stroke onset across studies. The number of patients ranged between 31 and 549 individuals, mean age between 55.7 and 73.9 years, and 49–72% of individuals were male (Table 1). Two studies included a healthy control group for comparison [22,24].

### 3.3. Quality Assessment

Quality appraisal according to ARHQ criteria revealed that the majority of studies, i.e., six investigations, were of high quality [22,24,25,27,28,29]. The two other studies included in this systematic review were of moderate quality [23,26] (Table 2).

### 3.4. Oral Health Records and Findings

Oral health parameters were rarely reported across studies. Four studies reported on missing teeth and/or denture status [22,23,24,25]. Three studies did not report on any oral parameters of the included studies [27,28,29]. Results of oral health in the included studies are presented in Table 3.

### 3.5. OHRQoL Measurements and Results

Five studies reported on the OHIP 14 for OHRQoL, showing means between 2.87 and 33.0 points in sum score [23,25,26,28,29]. Three studies applied GOHAI, with sum scores between 45.6 and 55.0 points [22,27,29]. One study used OHIP-EDENT to evaluate OHRQoL of the included patients and found a mean value of 18.8 points [24]. Out of the two studies investigating a healthy control group, only one study found worse OHRQoL in stroke patients [24]. Two studies reported on an association between OHRQoL and general health-related quality of life [28,29]. Three studies found OHRQoL to be associated with different oral health parameters [24,25,29], while two studies did not find an association [23,28]. Only one study found OHRQoL to be associated with stroke-related parameters [25], while three studies did not [22,24,28] (Table 4).

Only three studies reported on mean values for the different subscales/dimensions of OHRQoL measurement [24,25,26] (Table 5).

## 4. Discussion

This systematic review identified eight clinical studies, which investigated the OHRQoL of patients after stroke by different measurements. A healthy control group was only recruited by two studies, showing an ambivalent result [22,24]. Therefore, it will be necessary to discuss the applied instruments and the potential interpretation of the OHRQoL results first. The OHIP 14 is widely used across different research questions; thereby, it is a valid tool, which is very suitable for clinical studies [9]. The OHIP was developed in the early nineties, while different versions depending on the number of questions are available [30]. The OHIP reflects oral-health related issues, which patients perceived during the past month; thereby, questions are to be answered on a five-point Likert scale between 0 (never) and 4 (very often) [30]. Accordingly, a higher OHIP score indicates worse OHRQoL. While originally seven domains were defined within OHIP [31], recent research focused on four dimensions of OHRQoL, which are displayed by OHIP; those include oral function, psychosocial impact, oral pain, and orofacial appearance [32]. In absence of a healthy control group in the studies using the OHIP 14, the values can only be interpreted with regard to the literature. Overall, an OHIP 14 value between 0 and 6 points, depending on dentition (fully dentate up to toothless having full prosthesis), can be seen as “unaffected” OHRQoL [33]. Therefore, majority of studies indicated a reduced OHRQoL. The OHIP 14 values across the included studies varied between 2.87 and 33.0 points in sum score; this is a wider range as for other patient cohorts, e.g., rheumatoid arthritis, renal insufficiency, Alzheimer disease, or organ transplantation [16,17,18,19]. This argues for a certain heterogeneity of the included studies, which can be seen in Table 1 and will be discussed later below.

The second most common OHRQoL assessment was the GOHAI, which was applied in three studies. This index includes 12 questions related to oral conditions and is answered on a Likert scale, whereby answers range between 1 (never) and 5 (always) [34]. In contrast to OHIP, higher values indicate better OHRQoL. The GOHAI is widely used for elderly individuals or patients with dentures [35,36]. As the mean age of most studies was quite high in the included studies, the application of GOHAI appear reasonable. The study using healthy comparison for GOHAI did not find a difference between stroke survivors and healthy individuals [22]. Altogether, the GOHAI values in the current study are similar than for elderly individuals in literature [36,37], and were slightly better and showed smaller range across studies than for patients with rheumatoid arthritis, renal insufficiency, or Alzheimer disease [16,18,19]. Of course, this comparability is limited, because those diseases are different, but it might help to categorize the current reviews findings.

A slightly or moderately reduced OHRQoL of patients after stroke was previously hypothesized. Considering the abovementioned results, this hypothesis appears to be confirmed. Oral diseases, including tooth loss and periodontal diseases, can negatively affect OHRQoL [38,39]. It is known that patients after stroke show worse oral conditions, and periodontal diseases could even be related to stroke onset [14,15]. Although evidence is limited, a systematic review concluded stroke to be associated with tooth loss [40]. Four studies reported on tooth loss within the current review, showing remarkable number of missing teeth and high amount of denture wearers [22,23,24,25]. This might lead to the assumption that this issue would be an explanation for the reduced OHRQoL; however, only one study found associations between missing teeth and OHRQoL [25]. Considering the rarity of reported associations between oral health and OHRQoL across included studies, oral health situation might not be the main influential factor on OHRQoL in stroke patients. Nevertheless, this is limited by the fact that not all studies assessed oral health parameters and their potential relation to OHRQoL.

Against this background, other factors might influence the OHRQoL within the included studies. It is known that stroke is a life-changing event, potentially leading to disability and psychosocial complaints which affects quality of life of patients [4,5,6]. Severe general diseases can also influence the patients’ experience of their oral conditions; on the one hand, psychosocial complaints also affect this domain of OHRQoL, and on the other hand, general disease burden can mask oral complaints (response shift) [11,17]. Two studies found a relationship between OHRQoL and general health-related quality of life [28,29], supporting this assumption. Additionally, the disability and related motoric skills in stroke survivors might cause functional complaints related to the oral cavity, resulting in reduced OHRQoL. This is, however, not plausibly supported by the current systematic review, because one study found an association between OHRQoL and disability [25], while three did not [22,24,28]. Nevertheless, one other issue would argue for such an influence and concomitantly explain the high range of findings to some extent: the differences in time since stroke across included investigations. It can be seen from Table 1 that there was an enormous heterogeneity regarding this. Within the respective longitudinal studies, a certain positive time effect on OHRQoL is visible, which might indicate a positive effect of rehabilitation time on OHRQoL [27,28,29]. Rehabilitation is essential for patients after stroke to regain their independence and for a certain recovery of motoric functions [2,41,42]. Onward rehabilitation, especially physical activity, positively influences quality of life of individuals after stroke [41,42]. Therefore, it is not surprising that time since stroke might also influence OHRQoL of patients. Another factor, which potentially affects the OHRQoL across studies is the different mean age (see Table 1). Age is related to tooth loss and thus OHRQoL of individuals [38,43]. This is another factor that can explain the high range of OHRQoL outcomes in the current systematic review.

Altogether, the hypothesis that patients after stroke would show a slightly or moderately reduced OHRQoL, which is not primarily associated with their oral status, can be seen as partly confirmed. The major factors limiting the ability to support this hypothesis is the heterogeneity of included studies and the missing consideration of the respective parameters (oral health, quality of life, and disease-related parameters) in potential association with OHRQoL. This leads to the general limitations of this systematic review article. The heterogeneity across studies, regarding country, age, gender distribution, and time since stroke, limits the comparability of included studies. Similarly, the different assessment measurements of OHRQoL need to be recognized; although both OHIP and GOHAI are validated and appropriate for measuring OHRQoL, several differences exist between those measurements [44]. Furthermore, sub-scales of OHRQoL were rarely reported across included studies (see Table 5), making conclusions on the main reason for OHRQoL impairment (whether it is more functional or more psychosocial) speculative. In this context, the inhomogeneous consideration of general and disease-related parameters, general quality of life, and oral conditions limits the ability to draw conclusions on respective associations. Altogether, further studies are required, applying standardized and valid methodology and comprehensive assessment of OHRQoL and potential influential factors. Based on the included studies, the discussion limits the impact on oral health mostly to oral disease, which are related to oral hygiene and disease history (tooth loss and periodontal disease). In contrast, the most violent impact of stroke on the OHRQoL may be rather related to the instant facial palsy following stroke, possibly impairing motoric control, sensibility of the oral tissues, and chewing efficiency. This has been rarely reported, yet. Another factor with a potential psycho-social impact is the drooling of saliva, given that the lip closure might be affected by the facial palsy. Therefore, immediate functional impairment and a rather long-term effect regarding tooth loss and periodontal disease (which may have been present already before the stroke, and might have even contributed to its occurrence) should be distinguished and examined in subsequent studies. Future research in the field should therefore focus on the stroke-related functional and psycho-social issues and their impact on OHRQoL.

Finally, it must be discussed what might be the practical implication of the current study’s findings. Although included studies and their results were heterogeneous, a certain relevance of oral conditions and perceived oral health appears relevant for patients after stroke. Therefore, oral conditions and their potential impact on quality of life require consideration during rehabilitation and care after stroke. With regard to the association between worse oral conditions, periodontitis, tooth loss, and stroke [14,15,40], oral health should be fostered in this patient group, starting immediately in the rehabilitation of patients. Therefore, medical staff and caregivers should be sensitized for oral health issues and support oral hygiene and dental (prevention oriented) consultations. In dental context, patients appear to need an interdisciplinary approach, addressing all important risks and needs of the patients, e.g., as displayed in the concept of individualized prevention [45].

## 5. Conclusions

Studies on OHRQoL of patients after stroke are heterogeneous regarding design and results. Within these limitations, a reduced OHRQoL can be concluded for those individuals, with an unclear association to oral health, general quality of life, and disease-related parameters. Oral health issues should be recognized in patients after stroke, whereby medical staff and caregivers should support oral hygiene and dental visits, to foster oral health and OHRQoL in those individuals.

## Figures and Tables

**Table 1:** General information on the included studies. Values are presented as the mean values ± standard deviation, mean values (range) or percentages.

Author, Year	Country	No. of Patients	Status/Time after Stroke	Study Type	Subjects Mean Age in Years	Male (%)	Healthy Control Group for OHRQoL
McMillan et al., 2005 [22]	China	43	rehabilitation after stroke	monocentric cross-sectional	73.9 ± 6.1	60%	yes, n = 43, 44% male, age: 73.7 ± 6.1 years
Hunter et al., 2006 [23]	Scotland	41	1 year after stroke	monocentric cross-sectional	69 ± 9.8	49%	no
McGrath et al., 2009 [27]	China	acute stroke: 121, after 6 months: 65	acute stroke and 6 months later	monocentric observational with 6 months follow-up	acute stroke: 67.7 ± 11.1; after 6 months: 68.3 ± 11.3	acute stroke: 69%, after 6 months: 72%	no
Schimmel et al., 2011 [24]	Switzerland	31	hospitalized after stroke	monocentric cross-sectional	69.0 ± 12.7	58%	yes, n = 2 4, 54% male, age: 68.8 ± 10.8 years
Lam et al., 2014 [28]	China	81	rehabilitation after stroke (time since stroke onset 13 ± 6.8 days)	randomized clinical trial	69.9 ± 10.9	63%	no
Jang et al., 2015 [25]	Korea	549	care at home after stroke (0–10 years after stroke)	monocentric cross-sectional	<60 years: 13.8%, 60–69 years: 35.3%, 70–79 years: 40.5%, ≥80 years: 10.4%	60%	no
Dai et al., 2017 [29]	China	baseline: 94, after 3 months: 74	rehabilitation after stroke	randomized clinical trial	n/a	n/a	no
Lawal et al., 2020 [26]	Nigeria	60	onset less than 3 months: 10%, 3–6 months: 23.3%, >6 months: 66.7%	monocentric cross-sectional	55.7 ± 12.9	53.3%	no

OHRQoL: oral health-related quality of life; n/a: not applicable.

**Table 2:** Quality appraisal following the Agency for Healthcare Research and Quality (ARHQ) [21].

Item	McMillan et al., 2005 [22]	Hunter et al., 2006 [23]	McGrath et al., 2009 [27]	Schimmel et al., 2011 [24]	Lam et al., 2014 [28]	Jang et al., 2015 [25]	Dai et al., 2017 [29]	Lawal et al., 2020 [26]
(1) Define the source of information (survey, record review)	Yes	Yes	Yes	Yes	Yes	Yes	Yes	Yes
(2) List inclusion and exclusion criteria for exposed and unexposed subjects (cases and controls) or refer to previous publications	Yes	No	Yes	Yes	Yes	Yes	Yes	Yes
(3) Indicate time period used for identifying patients	Yes	Yes	Yes	Yes	Yes	Yes	No	Yes
(4) Indicate whether or not subjects were consecutive if not population-based	Yes	Yes	Yes	Yes	Yes	Yes	Yes	Yes
(5) Indicate if evaluators of subjective components of study were masked to other aspects of the status of the participants	No	No	No	No	Yes	No	Yes	No
(6) Describe any assessments undertaken for quality assurance purposes (e.g., test/retest of primary outcome measurements)	Yes	No	Yes	Yes	Yes	Yes	Yes	No
(7) Explain any patient exclusions from analysis	Yes	Yes	Yes	Yes	Yes	Yes	Yes	Yes
(8) Describe how confounding was assessed and/or controlled.	Yes	No	Yes	Yes	Yes	Yes	Yes	Yes
(9) If applicable, explain how missing data were handled in the analysis	NA	NA	NA	NA	NA	NA	Yes	NA
(10) Summarize patient response rates and completeness of data collection	Yes	Yes	Yes	Yes	Yes	Yes	Yes	Yes
(11) Clarify what follow-up, if any, was expected and the percentage of patients for which incomplete data or follow-up was obtained	NA	NA	Yes	NA	Yes	NA	Yes	NA
Total Score	8	5	9	8	10	9	10	7

**Table 3:** Oral health parameters and respective main results across included studies. Findings are given as the mean values ± standard deviation or percentages.

Author, Year	Tooth Loss, Remaining Teeth, Dentures	Dental Diseases, Caries, Dental Treatment Need	Oral Hygiene Indices	Periodontal Parameters, Periodontal Treatment Need	Further Oral Health Parameters
McMillan et al., 2005 [22]	number of teeth: 14.6 ± 10.8, M-T: 18.0 ± 10.7, removable denture: 40.5%, complete denture: 20.9 (maxilla), 23.3 (mandible)	D-T: 2.7 ± 3.7, F-T: 0.9 ± 2.1, DMF-T: 21.6 ± 9.7	n/a	n/a	n/a
Hunter et al., 2006 [23]	44% edentolous and wore full dentures, mean number of teeth in dentate: 17 teeth, 52% of dentate wear dentures	n/a	n/a	80% of dentate patients had CPI 1 or 2	n/a
McGrath et al., 2009 [27]	n/a	n/a	n/a	n/a	n/a
Schimmel et al., 2011 [24]	number of teeth: 18.8 ± 8.9, number of occlusal units: 4.3 ± 4.0, removable partial denture: 19.4%	n/a	n/a	n/a	colour mixing test (masticatory efficiency) 0.0901 ± 0.0488, maximum lip force: small: 5.29 ± 1.92 N, medium: 6.70 ± 2.88 N, large: 8.68 ± 4.13 N
Lam et al., 2014 [28]	n/a	n/a	n/a	n/a	n/a
Jang et al., 2015 [25]	number of missing teeth: 9.1%, 1–8 missing teeth: 24.5%, more than 9 missing teeth: 70.6%, denture use: 46.9%, denture not use: 19.5%	n/a	n/a	n/a	n/a
Dai et al., 2017 [29]	n/a	n/a	n/a	n/a	n/a
Lawal et al., 2020 [26]	n/a	n/a	OHI-S: male 2.42 ± 1.34, female: 2.22 ± 0.91	n/a	n/a

M-T: missing teeth, D-T: decayed teeth, F-T: filled teeth, DMF-T: decayed-, missing- and filled teeth index, CPI: community periodontal index, OHI-S: simplified oral hygiene index, and n/a: not applicable.

**Table 4:** Applied assessments for OHRQoL and relevant results for the included studies.

Author, Year	Assessment of OHRQoL	OHRQoL Worse than Healthy Control (HC)	Association/Correlation between OHRQoL and General HRQoL	Association/Correlation between OHRQoL and Oral Health	Association and/or Correlation between OHRQoL and Disease-Related Parameters
McMillan et al., 2005 [22]	GOHAI: 52.0 (48.0–57.0)	no, 54.0 (49.0–57.0)	n/a	n/a	no association
Hunter et al., 2006 [23]	OHIP 14, no sum score or mean reported	n/a	n/a	no association	n/a
McGrath et al., 2009 [27]	GOHAI: acute: 45.6 ± 8.5; after 6 months: 48.9 ± 6.9	n/a	n/a	n/a	n/a
Schimmel et al., 2011 [24]	OHIP-EDENT: 18.8 ± 15.5	yes, 12.3 ± 17.7	n/a	OHIP-EDENT associated with masticatory efficiency	no association
Lam et al., 2014 [28]	OHIP 14: baseline: 7.0 (2.0–14.0), after 3 weeks: 4.0 (1.0–9.0)	n/a	OHIP 14 associated to SF-12	no association	no association
Jang et al., 2015 [25]	OHIP 14: male: 33.0 ± 9.0, female: 33.1 ± 9.4	n/a	n/a	OHIP 14 associated with number of missing teeth	OHIP 14 associated with degree of disability and Barthel Index
Dai et al., 2017 [29]	GOHAI: baseline: 54.0 (49.0–56.0), follow-up: 55.0 (50.8–58.0), OHIP 14 baseline: 4.0 (2.0–12.0), follow-up: 3.5 (1.0-8.3)	n/a	GOHAI and OHIP 14 associated with PCS score of SF-12	GOHAI associated with plaque accumulation	n/a
Lawal et al., 2020 [26]	OHIP 14: 2.87 ± 0.78	n/a	n/a	n/a	n/a

n/a: not applicable, OHIP: oral health impact profile, PCS: physical compound summary, GOHAI: geriatric oral health assessment index, OHIP EDENT: oral health impact profile for denture wearers, and SF-12: short form 12 questionnaire.

**Table 5:** Subscales of OHRQoL in the included studies, if applicable. The results are given as the mean values ± standard deviation or otherwise as percentages.

OHIP 14							
Author, Year, Disease	Functional Limitation	Physical Pain	Psycho-Social Discomfort	Physical Disability	Psychological Disability	Social Disability	Handicap
Jang et al., 2015 (male/female) [25]	5.7 ± 2.0/5.9 ± 2.1	5.2 ± 1.8/5.4 ± 1.9	4.7 ± 1.5/4.6 ± 1.5	5.2 ± 1.8/5.1 ± 1.9	3.8 ± 1.4/3.7 ± 1.4	3.8 ± 0.4/3.6 ± 1.4	4.7 ± 1.8/4.7 ± 1.9
Lawal et al., 2020 [26]	0.65 ± 0.18	1.12 ± 0.27	0.63 ± 0.18	0.70 ± 0.19	0.27 ± 0.09	0.03 ± 0.02	0
**OHIP-EDENT**							
Schimmel et al., 2011 [24]	4.2 ± 3.7 *	4.2 ± 3.6 *	3.0 ± 2.8	3.1 ± 3.8	1.9 ± 2.1	0.8 ± 1.6	1.6 ± 2.1

OHIP: oral health impact profile. OHIP EDENT: oral health impact profile for denture wearers. * significant different from control.

## Supplementary Materials

The following are available online at https://www.mdpi.com/article/10.3390/jcm11051415/s1, Table S1: Excluded full-text articles screened for eligibility with reason for exclusion [46,47,48,49,50,51,52,53].

## Abbreviations

D-T	number of decayed teeth
DMF-T	decayed, missing, and filled teeth index
F-T	number of filled teeth
GOHAI	geriatric oral health assessment index
M-T	number of missing teeth
OHIP	oral health impact profile
OHRQoL	oral health-related quality of life
PICO	patient, intervention, control, outcome
