# Supplementary material for: Oral Health-Related Quality of Life in Patients after Stroke—A Systematic Review
Source: J Clin Med. 2022 Mar 4;11(5):1415. doi: 10.3390/jcm11051415 (PMC8911029; doi:10.3390/jcm11051415)
Supplement: Supplementary file 1 [file jcm-11-01415-s001.zip › jcm-1544214-supplementary.pdf]

Table S1. Excluded full-text articles screened for eligibility with reason for exclusion.

Author and Year	Reason for Exclusion
Zhu et al. 2008	No explicit reporting of stroke patients
Molania et al. 2021	No explicit reporting of stroke patients
Pengpid et al. 2021	No explicit reporting of stroke patients
Parat et al. 2020	No explicit reporting of stroke patients
de Bataille et al. 2021	No explicit reporting of stroke patients
Montero et al. 2021	No explicit reporting of stroke patients
Vasiliu et al. 2019	No explicit reporting of stroke patients
Fan et al. 2019	No explicit reporting of stroke patients

OHRQoL: oral health-related quality of life.

Supplementary Table S1 References

S1. Zhu HW, McGrath C, McMillan AS, Li LS. Can caregivers be used in assessing oral health-related quality of life among patients hospitalized for acute medical conditions? Community Dent Oral Epidemiol. 2008 Feb;36(1):27-33. doi: 10.1111/j.1600-0528.2006.00370.x. PMID: 18205637.

S2. Molania T, Malekzadeh Shafaroudi A, Taghavi M, Ehsani H, Moosazadeh M, Haddadi A, Gholizadeh N, Salehi M. Oral health-related quality of life (OHRQoL) in cardiovascular patients referring to Fatima Zahra Hospital in Sari, Iran. BMC Oral Health. 2021 Aug 11;21(1):391. doi: 10.1186/s12903-021-01756-0. PMID: 34380490; PMCID: PMC8356446.

S3. Pengpid S, Peltzer K. Prevalence and Associated Factors of Self-rated Oral Health among a National Population-based Sample of Adults in Sudan: Results of the 2016 STEPS Survey. Oral Health Prev Dent. 2021 Jan 7;19(1):391-397. doi: 10.3290/j.ohpd.b1749751. PMID: 34259432.

S4. Parat K, Radić M, Perković D, Lukenda DB, Kaliterna DM. Reduced salivary flow and caries status are correlated with disease activity and severity in patients with diffuse cutaneous systemic sclerosis. J Int Med Res. 2020 Oct;48(10):300060520941375. doi: 10.1177/0300060520941375. PMID: 33081544; PMCID: PMC7588767.

S5. de Bataille C, Castellan M, Massabeau C, Jouve E, Lacaze JL, Sibaud V, Vigarios E. Oral mucosal changes induced by adjuvant endocrine therapies in breast cancer patients: clinical aspects and proposal for management. Support Care Cancer. 2021 Apr;29(4):1719-1722. doi: 10.1007/s00520-020-05797-z. Epub 2020 Nov 2. PMID: 33140247.

S6. Montero J, Dib A, Guadilla Y, Flores J, Pardal-Peláez B, Quispe-López N, Gómez-Polo C. Functional and Patient-Centered Treatment Outcomes with Mandibular Overdentures Retained by Two Immediate or Conventionally Loaded Implants: A Randomized Clinical Trial. J Clin Med. 2021 Aug 6;10(16):3477. doi: 10.3390/jcm10163477. PMID: 34441773; PMCID: PMC8396916.

S7. Vasiliu MP, Sachelarie L, Popovici D, Farcas DM. Oral Health-Related Quality of Life in the Case of Elderly People from Romania Before and After the Prosthetic Treatment. Int J Med Res Health Sci 2019;8:33-39

S8. Fan WY, Tiang N, Broadbent JM, Thomson WM. Occurrence, Associations, and Impacts of Nocturnal Parafunction, Daytime Parafunction, and Temporomandibular Symptoms in 38-Year-Old Individuals. J Oral Facial Pain Headache. 2019 Summer;33(3):254–259. doi: 10.11607/ofph.2221. Epub 2018 Oct 26. PMID: 30371683.
